# A Triboelectric Nanocomposite for Sterile Sensing, Energy Harvesting, and Haptic Diagnostics in Interventional Procedures from Surgical Gloves

**DOI:** 10.1002/adhm.202202673

**Published:** 2023-03-11

**Authors:** Carmen Salvadores Fernandez, Shireen Jaufuraully, Biswajoy Bagchi, Wenqing Chen, Priyankan Datta, Priya Gupta, Anna L. David, Dimitrios Siassakos, Adrien Desjardins, Manish K. Tiwari

**Affiliations:** ^1^ Nanoengineered Systems Laboratory Mechanical Engineering University College London London WC1E 7JE UK; ^2^ Wellcome/EPSRC Centre for Interventional and Surgical Sciences University College London London W1W 7TS UK; ^3^ Elizabeth Garrett Anderson Institute for Women's Health University College London London WC1E 6AU UK; ^4^ NIHR Biomedical Research Centre at UCL London W1T 7DN UK; ^5^ Department of Medical Physics and Biomedical Engineering University College London London WC1E 6BT UK

**Keywords:** multifunctional nanocomposite sensors, nanocomposites, self‐powered sensors, stiffness detection, triboelectric sensors

## Abstract

Advanced interfacial engineering has the potential to enable the successful realization of three features that are particularly important for a variety of healthcare applications: wettability control, antimicrobial activity to reduce infection risks, and sensing of physiological parameters. Here, a sprayable multifunctional triboelectric coating is exploited as a nontoxic, ultrathin tactile sensor that can be integrated directly on the fingertips of surgical gloves. The coating is based on a polymer blend mixed with zinc oxide (ZnO) nanoparticles, which enables antifouling and antibacterial properties. Additionally, the nanocomposite is superhydrophobic (self‐cleaning) and is not cytotoxic. The coating is also triboelectric and can be applied directly onto surgical gloves with printed electrodes. The sensorized gloves so obtained enable mechanical energy harvesting, force sensing, and detection of materials stiffness changes directly from fingertip, which may complement proprioceptive feedback for clinicians. Just as importantly, the sensors also work with a second glove on top offering better reassurance regarding sterility in interventional procedures. As a case study of clinical use for stiffness detection, the sensors demonstrate successful detection of pig anal sphincter injury ex vivo. This may lead to improving the accuracy of diagnosing obstetric anal sphincter injury, resulting in prompt repair, fewer complications, and improved quality of life.

## Graphical abstract

Novel sprayable multifunctional triboelectric sensors are exploited as antibacterial, superhydrophobic, nontoxic, ultrathin tactile sensors. They are directly integrated on the fingertips of surgical gloves and work with a second sterile glove on top. The sensors enable mechanical energy harvesting, force sensing, and detection of material's stiffness changes. Finally, they demonstrate successful detection of pig anal sphincter injury ex vivo.

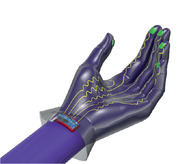

## 1 Introduction

Sensorized gloves and fingertip‐mounted sensors have tremendous potential in a variety of healthcare engineering applications such as robotic interventions, prosthetics, and human–machine interfaces.^[^ ^1^, ^2^ ^]^ Haptic sensors concentrating on mechanical stimuli such as pressure, force, and mechanical vibrations^[^ ^3^ ^]^ are the most widely explored.^[^ ^4^ ^]^ Sensorized gloves also offer attractive benefits for use as a biosensing platform for diagnostics and food safety applications.^[^ ^5^ ^]^ A majority of these applications are served with the sensing hardware exploiting a battery or other portable power source for powering or in simple cable‐tethered mode. However, recent works have also made progress in investigating sensing mechanisms such as those using triboelectric sensors that enable harvesting of otherwise wasted mechanical energy for self‐powering.^[^ ^6^ ^]^ A recent work from Wang's group has introduced the idea of harvesting energy using electrochemical means to develop self‐powered sensors that can be mounted on the fingertip.^[^ ^7^ ^]^ The approach offers an additional benefit as the thinness of these fingertip‐mounted external sensors is invaluable to avoid interfering with the sensory perception of the user.^[^ ^8^ ^]^ This is a major requirement for the use of such sensors in interventional healthcare applications where a clinician is involved.

Integration of sensors on flexible, surgical gloves should also meet a number of additional key requirements. First, an inherent ability to prevent contamination by dirt or impurities such as those achieved by self‐cleaning (e.g., superhydrophobic) triboelectric materials is promising here, though the number of works demonstrating self‐cleaning, glove‐integrated sensors is limited. Second, the sensors and their components need to maintain sterility. Antimicrobial nanocomposites offer a promise to this end;^[^ ^9^ ^]^ however, flexible, glove‐integrated antimicrobial sensors remain to be introduced. In fact, concerns about sterility may be avoided if the sensors are still functional even when mounted on a glove underneath a second sterile glove worn on top. Such double glove use is commonplace in interventional procedures and the approach thus may facilitate a rapid translation from laboratory to clinic. Third, sensor integration on a surgical glove is an important challenge and has multiple fabrication steps. For example, making sensors followed by integration on to gloves is time consuming and also opens up room for faults. Scalability of the fabrication approach is another, related concern. In fact, an ideal sensing platform needs to cater to all these requirements simultaneously and remains to be realized.

**Figure 1:**
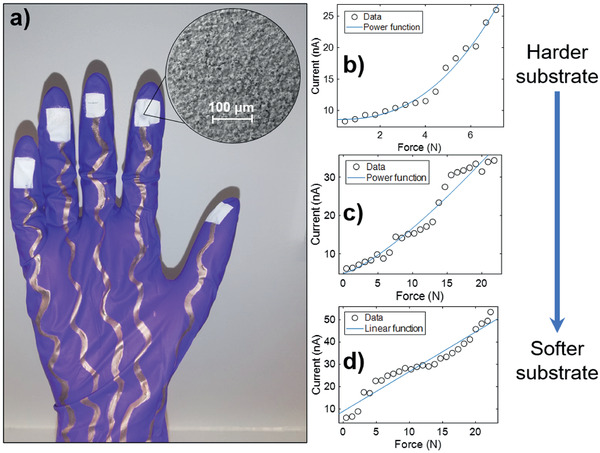
a) Photograph of the sensorized surgical glove prototype. Outputs of triboelectric sensors applied on substrates with different stiffness, b) glass, c) Polydimethylsiloxane (PDMS)_ (mixing ratio 10:1 elastomer to curing agent), and d) ecoflex. Each fitting curve had *R*‐value of ≥0.94.

To meet the above requirements, here we introduce a sprayable nanocomposite as a triboelectric force sensing layer that can be sprayed directly onto printed electrodes on surgical gloves (**Figure** **1**a). Designing such sensors out of nanocomposites may offer numerous advantages. First, the nanocomposites offer a wide choice of easily accessible, low‐cost polymers that lends themselves to simple processing and can serve as a suitable matrix for the nanocoatings and sensors.^[^ ^10^ ^]^ Second, nanocomposites can be applied—just as coatings or films—on essentially any surface, as long as adhesion is managed.^[^ ^11^ ^]^ Finally, the introduction of nanoparticle fillers, which are key to control surface roughness, can enable the tuneable inclusion of additional properties,^[^ ^12^ ^]^ i.e., it can help add the multifunctionality outlined above. In fact, simple spray‐coated nanocomposite coatings readily impact two important properties; antibacterial activity and superhydrophobicity. These coatings can prevent biofouling, infection, and moisture contamination that are particularly important to avoid in interventional healthcare applications, since the vast majority of biofilm‐associated infections are contracted in a hospital.^[^ ^13^, ^14^ ^]^ In addition, bacterial infections and biofilms are also linked to the increasingly worrying trend of antimicrobial resistance (AMR), because the bacteria evolve and are able to withstand the effect of medication designed to eradicate them. The initial contact with the bacteria can take place through the skin of either the healthcare worker or the patient, or through other means such as contaminated fluids.^[^ ^15^ ^]^ This raises the possibility of infection and biofilm formation. Biofilms on catheters or other medical devices can result in bacteremia—clinicians handle these devices (with gloves)—thus any sensors to be integrated on gloves must comply with the sterility requirements, and ideally prevent/mitigate sources of infections.

Superhydrophobic surfaces show promising anti‐fouling properties, and can inhibit adherence and the initial attachment of bacteria.^[^ ^16^, ^17^ ^]^ Previous works have showed that a rational selection of antibacterial nanoparticles not only renders the nanocomposite superhydrophobic, and thus antifouling, but also bactericidal.^[^ ^18^ ^]^ This implies that even if microbes manage to adhere despite the antifouling nature of the coating, they will be deactivated.^[^ ^19^ ^]^ As an important extension, here we demonstrate a multifunctional nanocomposite, which in addition to featuring infection and adhesion control properties offers the sensing and energy harvesting capabilities using the triboelectric effect.

Triboelectricity is a type of contact electrification that takes place when two dissimilar materials come into contact or are separated.^[^ ^20^, ^21^ ^]^ This contact electrification, together with electrostatic induction, enables triboelectric sensors to produce a current when they come into contact or are separated/rubbed against an object.^[^ ^22^, ^23^ ^]^ Triboelectric sensors can be self‐powered due to their energy harvesting capabilities,^[^ ^24^ ^]^ i.e., they can serve as a triboelectric nanogenerator (TENG).^[^ ^25^, ^26^ ^]^ TENGS have multiple operation modes: vertical contact‐separation, lateral sliding, single‐electrode, and freestanding triboelectric‐layer.^[^ ^27^ ^]^ Furthermore, most current TENGs are flexible (polymer‐based), effectively portable, and can be realized through inexpensive fabrication processes.^[^ ^28^ ^]^ TENG‐based sensors are suitable for static and low‐frequency dynamic pressures which enables them to detect different modes of contact, for example, during tapping and rubbing.^[^ ^6^ ^]^ In addition, they can be integrated with piezoelectric nanogenerators (PENGs) to enable wide bandwidth energy harvesting.^[^ ^29^ ^]^ Recently, Peng et al.^[^ ^30^ ^]^ developed a biodegradable antibacterial e‐skin comprising of a nanofibre‐based triboelectric nanogenerator, which achieved real‐time monitoring of physiological signal and joint movements. Jo et al.^[^ ^31^ ^]^ introduced a skin‐attachable motion sensor that was antibacterial and paper‐based. To this end, our sprayable nanocomposite offers self‐cleaning properties and ready integration directly on the substrate of choice, e.g., the flexible surgical gloves presented herein, and as such, this is the first demonstration of such multifunctionality.

Our triboelectric single‐electrode/coating‐based sensors allowed us to readily sense the tactile forces during normal manual contact/rubbing and demonstrate an ability to detect stiffness change. Unlike previous works that relied on tapping signals,^[^ ^32^ ^]^ we demonstrate the feasibility of detecting stiffness changes from rubbing signals using sensorized gloves which may be more suitable for future uses in interventional applications where a clinician is often space constrained. This is particularly useful for procedures where imaging/vision‐based approaches are difficult to use and tactile feedback is an attractive alternative. The tactile force sensing and stiffness change detection is also feasible with a second sterile glove on top. As a specific example of clinical application, such sensorized gloves could be used for vaginal and rectal examination and the resulting tactile signals could aid diagnosis and assessment of maternal anal sphincter injuries after vaginal birth. This is particularly important as up to 12% of women can sustain such an injury, which can have long‐lasting, devastating effects on quality of life.^[^ ^33^ ^]^ Effects include fecal incontinence, recurrent urinary tract infection, and fistula formation.^[^ ^34^ ^]^ Swift diagnosis and repair at the time of injury is required in order to restore normal anatomy. Nearly 40% of obstetric anal sphincter injuries are missed on primary assessment, and, if missed, can lead to worsening fecal incontinence.^[^ ^35^ ^]^ To establish the proof of concept, we demonstrate that the rubbing signal from our sensorized surgical gloves can accurately detect injury in a porcine anal sphincter model.

## 2 Results and Discussion

### 2.1 Triboelectric Properties and Force Calibration

Figure 1a shows a typical glove with screen‐printed silver electrode/interconnect and sprayed nanocomposite triboelectric coatings at each of the five glove fingertips. To characterize the triboelectric properties, we first tested the coating as a standard freestanding triboelectric‐layer.^[^ ^36^ ^]^ The triboelectric signals from the coating were recorded using a spring‐loaded board (S1), with an electrode separation of 3 mm. Current peaks were created when rubbing from side to side of the electrodes as shown in Figure S1 (Supporting Information) proving its triboelectric nature.

Output current from triboelectric sensors increases with the applied force, up to a threshold due to the limited deformability of the material.^[^ ^6^ ^]^ However, by spraying the triboelectric coating on different substrates with varying stiffnesses, we were able to tune the measurable force range. The harder the substrate, the lower the measurable force range achieved by the sensor (Figure 1b–d). Each sensor was calibrated individually by plotting the sensor output current against force values applied. The resulting data were fitted using either power law or linear polynomial. For Figure 1d, the linear fit yielded a high *R* ^2^ value of 0.94. These trends are consistent for similar triboelectric sensors in literature where linear and power function fit are typical.^[^ ^6^, ^37^, ^38^ ^]^ With our emphasis on interventional healthcare, we focused on the range of 0–20 N. The typical force applied across general surgery is 4.67 N (mean of average) and 11.4 N (mean of maximum),^[^ ^40^ ^]^ while for obstetrics specifically they are 8.69 N (mean of average) and 10.1 N (mean of maximum).^[^ ^40^ ^]^

The initial slope of the fitted curves (see Figure 1b–d) increases as the substrate becomes softer (1.78 nA N^−1^ for ecoflex, 1.04 nA N^−1^ for PDMS, and 0.93 nA N^−1^ for glass). This may be due to the decrease in deformation (and thus the contact area) with increasing stiffness. As a material becomes softer, it deforms more for a given applied force, resulting in a larger slope. This realization enabled us to detect stiffness change, as will be explored further in Section 2.6.

### 2.2 Coating Characterization

**Figure 2:**
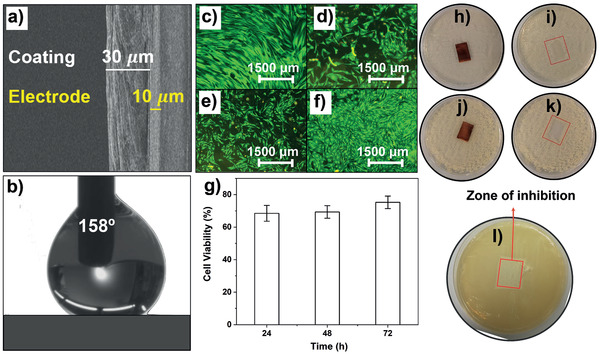
a) SEM image of the cross‐section of the triboelectric sensor, showing the thicknesses of the coating and the electrode. b) Picture of a water drop on the triboelectric coating, with *θ*_A_ of 158° and Δ*θ* of 6°. c–f) Fluorescence microscopy images showing c) cultured control Human Dermal Fibroblast (HDF) cells (without sample) and (d), (e), and (f) are cultured HDF cells treated with samples for 24, 48, and 72 h extracts respectively. Live cells are stained green and dead cells red. g) MTT assay results showing 75% ± 4% HDF cell viability after 72 h. h–l) Kirby–Bauer antibacterial test results showing no growth of neither *S. aureus* (h,i) nor *E. coli* (j,k) in the area where the sensor was placed. l) Kirby–Bauer antibacterial test results showing no growth of *S. agalactiae* (Group B streptococcus) in the area in which the sensor was placed (constituting the zone of inhibition).

The nanocomposite coating is formulated to be antibacterial, superhydrophobic, triboelectric, and noncytotoxic. The coating thickness was measured using scanning electron microscope (SEM) imaging (**Figure** **2**a). All the constituent materials are biocompatible and non‐toxic to human tissue following European Union material regulations (S2). The superhydrophobicity of the coatings (Figure 2b) is obtained due to the low energy of the polymers together with the hierarchically rough morphology created by the ZnO nanoparticles, as can be seen in the inset of Figure 1a.^[^ ^41^ ^]^ The hierarchical roughness of our coatings not only enhances the anti‐wetting characteristics^[^ ^42^ ^]^ but also the contact area and thus triboelectrification.^[^ ^43^ ^]^ Furthermore, surface roughness can prevent full contact between the electrode and the coating, since some areas remain untouched, making it more sensitive to the applied force. The wettability was characterized by measuring dynamics contact angles, which showed an advancing contact angle *θ*_A_ of 158° (Figure 2b) and contact angle hysteresis Δ*θ* of 6° that confirm superhydrophobicity and self‐cleaning properties.^[^ ^41^ ^]^ Energy‐dispersive X‐ray spectroscopy (EDS) images showing the distribution of elements in the coating are shown in Figure S2 (Supporting Information).

### 2.3 Cytotoxicity Tests

In order to assess biocompatibility, human dermal fibroblast (HDF) cell line was used as it is associated with tissue repairing and wound healing.^[^ ^44^, ^45^ ^]^ Fluorescence imaging (Figure 2c‐f) of HDF cells treated for different amounts of time, as described under Experimental Section (see section Cytotoxicity Tests), with extracts of the sensor material (coating) shows live cells (indicated by green fluorescence) with morphology characteristic of healthy HDF cells.^[^ ^46^ ^]^ Compared to control (Figure 2c), HDF cells treated with extracts for 24 h (Figure 2d) and 48 h (Figure 2e) show a slightly smaller number of live cells, but the cell number significantly recovers with 72 h extract (Figure 2d). This may be due to the agglomeration of released ZnO particles in the 72 h extracts. As would be expected, a higher number of ZnO nanoparticles would have been released throughout the 72 h period compared to the 24 or 48 h period, resulting in larger‐sized particle agglomerations. Being larger in size they produce less reactive oxygen species (ROS) and also are not able to penetrate cells, hence showing low cytotoxicity.^[^ ^47^ ^]^ HDF cell viability was further investigated by MTT assay (Figure 2g), and as expected the percentage of viable cells increased to 75% ± 4% when treated with 72 h extract.

### 2.4 Antibacterial Properties

ZnO is well known for its biocidal activity, which does not promote microbial resistance.^[^ ^47^ ^]^ The antibacterial activity of ZnO has been linked to several simultaneously active mechanisms.^[^ ^48^, ^49^ ^]^ First, ROS generation leads to oxidative stress on the bacteria, second, cell walls are damaged due to ZnO‐localized interaction and third, there is membrane disruption and permeability. Finally, the toxicity is caused by the dissolved Zn ions in the bacterial cytoplasm.^[^ ^50^ ^]^ As a result, the ZnO nanoparticles render the coating antibacterial and have been shown to deactivate *S. aureus* (ATCC 43300—Gram positive), *E. coli* (ATCC 25922—Gram negative), *S. agalactiae* (Gram positive—group B streptococcus that is commonly associated with maternal and neonatal sepsis^[^ ^51^ ^]^ and relevant for our interventional healthcare case study covered in Section 2.7). As evident from Figure 2h‐l, all the sensor materials show clear zones of inhibition for *E. coli* (Figure 2h,i), *S. aureus* (Figure 2j,k), and *S. agalactiae* (Figure 2l), which indicates no bacterial growth in the region where the coating is placed. It is also important to note that the zone of inhibition does not spread beyond the sample area, and this is most probably because ZnO nanoparticles are embedded in the coating and impart antibacterial activity through contact inhibition by ROS production.^[^ ^48^ ^]^ Thus, overall, the sensor material shows excellent antibacterial activity and good biocompatibility, which suggest the safe use of coatings (sensors) in healthcare application. As we will show below, during the actual operation, for enhanced sterility, the sensorized glove would be covered with a second surgical glove on top, which eliminates direct contact of sensor components with human tissue.

### 2.5 Energy Harvesting

**Figure 3:**
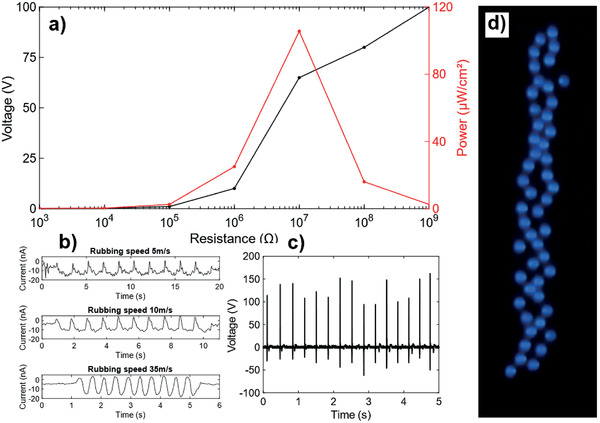
Energy generation characteristics of the triboelectric sensors. a) Output power density at different loads. b) Rubbing test at different speeds. c) Open‐circuit voltage. d) Photograph of LEDs powered by harvested energy.

As shown in **Figure** **3**a, a maximum output power density of 106 µW cm^−2^ is obtained at a load resistance of 10 MΩ (please see section Energy Harvesting Tests regarding the protocol and sensor geometry used in Experimental Section). Figure 3b shows the processed triboelectric signal from rubbing tests using the fingertip‐integrated sensors and the positioning set‐up described under Experimental Section (see Triboelectric Sensing/Detecting Setup). The current peaks are negative and remain unaffected, at about −10 nA, by the rubbing speed; the signal peak width corresponded to the time of the rubbing. Next, manual contact‐separation mode measurements were performed and yielded an open circuit voltage of up to 150 V, as shown in Figure 3c. Varying electrical loads were connected to the TENG sensors to measure maximum power out. The output of the triboelectric nanocomposite coating could be used to light up to 50 commercial blue light emitting diodes (LEDs) (Figure 3d; Video S1, Supporting Information). This shows the potential of creating a fully self‐powered tactile system in the future. In addition, the coatings can be readily sprayed on piezoelectric films to enhance their power output and bandwidth (while also rendering them antimicrobial and superhydrophobic) as described in Supporting Information S4. This attests to the scalability of our sprayed nanocomposite, which is suitable for use in nearly any flexible electronics platform^[^ ^53^ ^]^ by simply spray coating on a conductive surface.

### 2.6 Detecting Stiffness Change Using the Sensorized Surgical Glove

**Figure 4:**
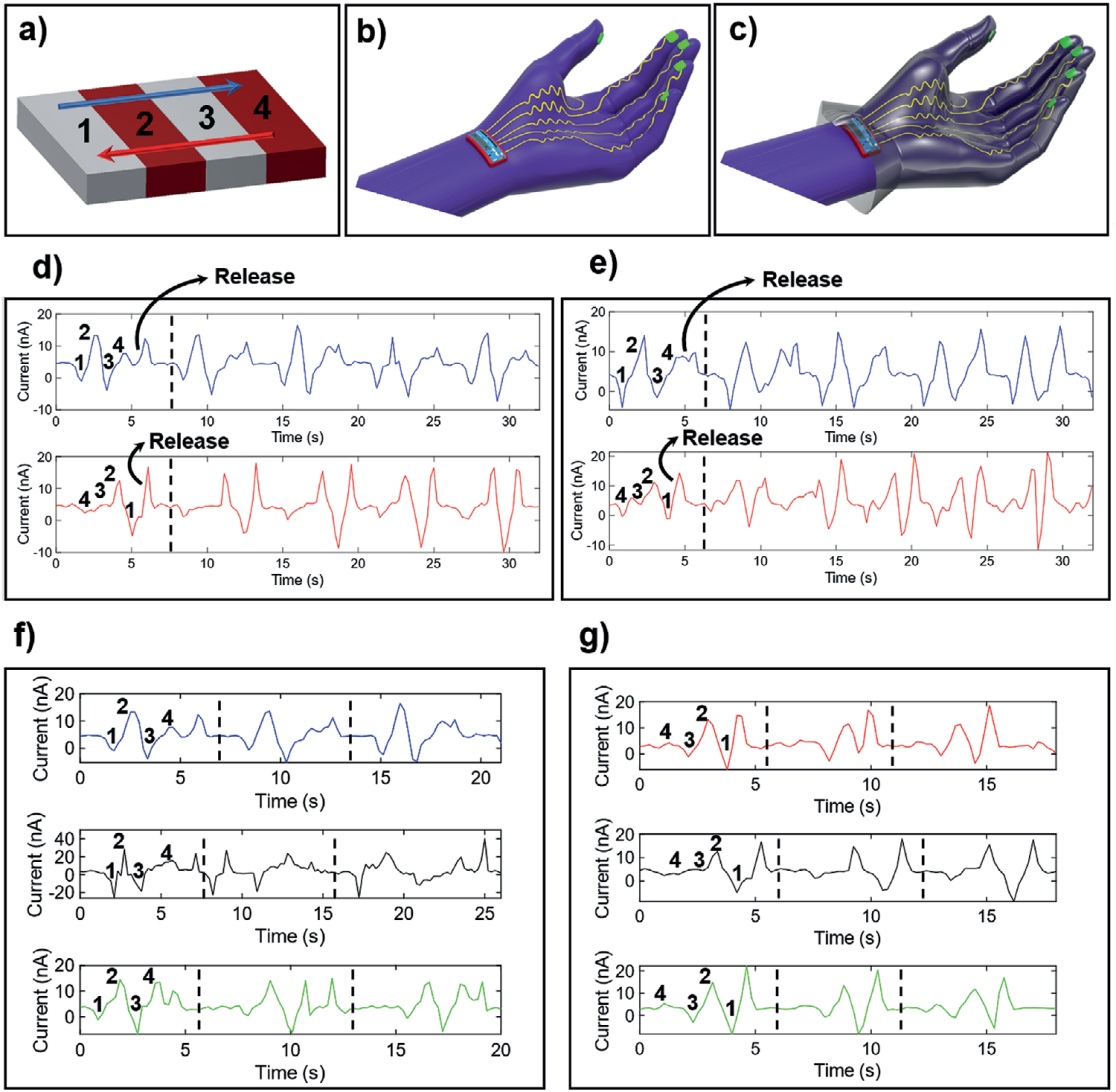
a) Schematic representation of the slots setup for the elastography tests (1 and 3 correspond to ecoflex and 2 and 4 to PDMS). b) Schematic illustration of the fingertip‐based tactile sensor glove for surgical applications. c) Schematic illustration of the fingertip‐based tactile sensor glove for surgical applications covered up by a second surgical glove on top. d) Comparison of peaks produced by rubbing against the 4 slots shown in (a) using the sensorized glove (from left to right, blue signal; from right to left, red signal). e) Comparison of peaks produced by rubbing against the four slots shown in (a) using the sensorized glove covered up by a second surgical glove (from left to right, blue signal; from right to left, red signal). f) Three different tests using three different glove samples showing the peaks produced by rubbing against the four slots shown in (a) using the sensorized glove from left to right. g) Three different tests using three different glove samples showing the peaks produced by rubbing against the four slots shown in (a) using the sensorized glove from right to left.

Following the stiffness‐dependent calibration in Section 2.1, a number of tests were carried out to assess the ability of the nanocomposite sensors to distinguish changes in stiffnesses in different materials, performed both manually and using the controlled motorized positioning setup (S5). **Figure** **4** shows the results to this end. The sensor signals were recorded by rubbing across 4 slots filled with materials of different stiffness (Figure 4a) directly with a sensorized glove (Figure 4b) and a sensorized glove covered by a surgical glove on top (Figure 4c). The thickness of the materials in each of the four slots was kept the same to ensure repeatability. Figure 4a depicts the four slots used, 1 and 3 correspond to ecoflex (“softer”) and 2 and 4 correspond to PDMS (“harder”). The resulting signal recordings are plotted in Figure 4d,e and show the distinctive peaks that form when going from one material to another, irrespective of the sensors being exposed or covered by an extra surgical glove. In these tests, negative peaks form when the sensor makes contact with a given material, and similarly positive peaks are formed when contact is released from the material (Video S2, Supporting Information). The fact that a current peak will be created when separating from the material with which the sensor has exchanged charges through previous contact is well established in literature.^[^ ^21^, ^53^, ^54^ ^]^ In turn, current peaks created when contacting a material for the first time have also been reported and seem to be due to the contribution of flexoelectricity.^[^ ^32^, ^55^ ^]^ Mechanical deformations caused by the contact or rubbing motion can lead to flexoelectric coupling^[^ ^56^, ^57^ ^]^ that is reflected by the first current peak when contacting slot 1 (or slot 4 in the reversed direction movement).

The peaks are dependent on the either increased or decreased contact area and the creation of strain gradients associated with the coating's deformation that takes place when variations in material stiffness are encountered, that is, when going from slot to slot (PDMS to ecoflex, or ecoflex to PDMS). The higher or lower deformation depending on the stiffness of a material results yields different sensor output.

When going from ecoflex to PDMS (such as from 1 to 2 in the blue signals or from 3 to 2 in red signals in Figure 4a,b), there is a decrease in contact area due to decreased deformation fields in PDMS relative to ecoflex, thus resulting in an effective “released contact”. When going from PDMS to ecoflex (such as from 2 to 3 in blue signals or from 2 to 1 in red signals in Figure 4a,b), there is an increase in contact area due to increased deformation fields in ecoflex relative to PDMS, thus resulting in “new contact”. Due to the surface roughness of the coating, when crossing from a harder material to a softer one, the softer material is able to conform to the coating filling up all the microscale “air gaps”. TENGs that rely on changes in the contact area to produce alternating current (AC) current outputs have similarly been reported.^[^ ^58^ ^]^ As a result of mixed triboelectric and flexoelectric charge transfer at the interface between the rough coating and the slots, we can observe that charge transfer is reversed with material strain, when both effects are in opposite directions.^[^ ^59^ ^]^

Finally, when contact is released, a current peak is always created in the positive y‐axis direction. This is due to contact separation with a material that is triboelectrically more negative than the material of the sensor.^[^ ^62^ ^]^

Figure 4f,g shows three repetitions of the aforementioned tests, in which we can once again see the trend described. The use of tactile sensors for the detection of changes in stiffness such as the ones seen in Figure 4 could be invaluable for a number of clinical applications.^[^ ^63^ ^]^ An example of such is explored in Section 2.7 as a case study. Another application can also be found in Videos S3 and S4 (Supporting Information), in which we used the sensorized surgical glove to facilitate accurate determination of fetal position.

### 2.7 Ex Vivo Detection Of Anal Sphincter Defects

**Figure 5:**
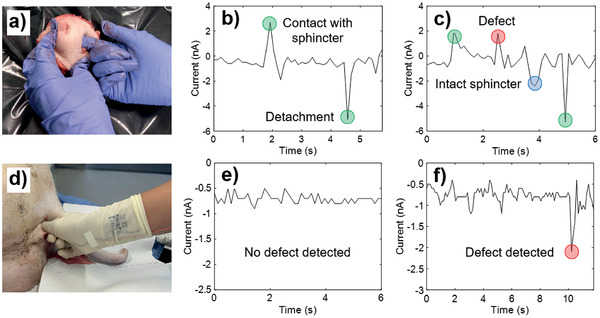
a) Obstetrician carrying out the test on the dissected ex vivo pig anal sphincter wearing the sensorized glove covered by a second surgical glove. b) Control test results on intact dissected anal sphincter. c) Test results showing accurate detection of the anal sphincter defect in the dissected sphincter. d) Obstetrician carrying out the test on an anal sphincter in a non‐dissected whole pig cadaver wearing the sensorized glove covered by a second surgical glove. e) Control test results on intact non‐dissected anal sphincter. f) Test results showing accurate detection of the defect in the non‐dissected anal sphincter.

For these tests, the sensorized gloves were covered by a second sterile surgical glove that is routinely used in surgical practice. An obstetric trainee with 6 years experience tested the sensorized glove first ex vivo on an intact and cut pig anal sphincter (**Figure** **5**a) and then in a whole pig cadaver (Figure 5b). This was performed by rubbing across the sphincter from side to side to demonstrate detection of the sphincter defect and as described in the protocol under Experimental Section (see section Pig Anal Sphincter Test Protocol). As shown in Figure 5b,c, the sensors on the index fingertip produce repeatable and distinguishable peaks when passing through the defect (the area which was cut) in the dissected sphincter, and when coming back in contact with the intact sphincter after the defect is traversed, due to the difference in stiffness and contact (following the mechanism described in Section 2.6). For the sphincter tests on the pig cadaver, the results once again show a distinguishable peak when crossing the defect (Figure 5e,f). For the latter tests, the obstetrician always kept the sensorized glove in contact with the sphincter due to limited space for maneuverability, which is why the peaks corresponding to “contact with sphincter” and “detachment” are not observed in the results. These results suggest the same trend and behavior of the sensors as that shown in Section 2.6, which served as the foundation to pursue this clinical application‐based case study. In these tests, positive peaks form when the sensor makes contact with the sphincter (as opposed to negative peaks such as the ones seen in Section 2.6, which is due to the relative positions of the material in contact with the sensor and the sensor itself in the triboelectric series), and similarly negative peaks are formed when contact is released from the material. When rubbing from the intact sphincter to the defect (where the sensor comes in contact with the softer anal mucosa at the base of the defect) the peak formed is in the positive y‐direction, due to the increased contact, deformation, and strain gradients associated with it. When going back from the defect to the intact sphincter, a negative peak is formed, due to decreased deformation fields in the intact sphincter relative to the softer anal mucosa beneath the defect. Notably, in the tests carried out on the non‐dissected sphincter in the whole pig cadaver, the peak produced when encountering the defect is always in the negative direction. This may seem counter‐intuitive at first, based on our previous rationale since the anal mucosa beneath the sphincter defect is softer than the sphincter muscle. Our interpretation is that the aforementioned lack of space and maneuverability, together with the finer cut created in the sphincter to produce the defect, resulted in the creation of a “slot” in the sphincter and a small air gap between the sensor (together with the obstetrician's fingertip) and the skin. Stiffness is no longer the governing factor affecting the induced current, since when the defect is crossed, the sensor is unable to fully make contact with the softer anal mucosa underneath and instead is subject to contact separation due to the small gap (“slot”) created instantaneously when rubbing through the sphincter.

The obstetrician repeated these tests 30 times for each scenario (dissected intact sphincter, dissected sphincter with a defect, non‐dissected intact sphincter and non‐dissected sphincter with a defect). As can be seen in the box plots in Supporting Information S6, the sensor achieves 100% sensitivity and 100% specificity in detecting the sphincter defect for the non‐dissected pig cadaver tests. As shown in Video S5 (Supporting Information), a simple user‐friendly interface set up using LabView is able to detect the defect by simply specifying a threshold current level (−1.5 nA) below which the peak created corresponds to the defect (clearly visible in the boxplot in Supporting Information S6). Ultimately, the sensors produce repeatable and distinguishable peaks when going through the defect in the sphincter, leading to the successful detection of the injury. Additionally, due to the thinness achieved by integrating the coating directly onto the surgical glove, clinicians’ sensory perception remained unaffected, which is critical toward its effective integration in the clinical workflow.^[^ ^62^ ^]^ It is also crucial to emphasize the importance of the adhesion of the coating to the surgical gloves. The adhesion performance and any undesirable coating delamination caused by mechanical stress will affect the performance, durability, and safety of the sensors.^[^ ^65^ ^]^ The method of applying the coating was thoroughly investigated and standardized in order to maintain both its desirable thinness and robustness (Supporting Information S7). Tape peel test was carried out to ensure satisfactory adhesion of the coating to the surgical gloves (S8 and Video S6, Supporting Information). Although unnecessary for the applications considered herein and beyond the scope of the current work, further improvements may also be realized through the incorporation of biocompatible adhesion promoters.^[^ ^66^ ^]^

## 3 Conclusion

In this work, an easy‐to‐fabricate, cost‐effective triboelectric nanocomposite coating has been exploited, with both sensing and energy harvesting capabilities. Integrating the nanocomposite into surgical glove fingertips or other typical clinical devices has strong potential for a wide range of clinical applications by enabling real‐time force readings and detection in changes in stiffness of tissues. A surgical glove with fingertip‐based ultrathin sensors is likely to be favorable from the standpoint clinical translation; a glove identical to the one worn in usual procedures and surgeries could be used, with the prominent addition of embedded coatings with sensing capabilities. Due to the superhydrophobic and antibacterial nature of the triboelectric nanocomposite, biofouling and biofilm formation will be avoided. Furthermore, the electrical outputs of the proposed glove‐based health monitors will not suffer from fluctuations upon changes in environmental conditions such as temperature and air humidity which will lead to maintained accurate sensing. Additionally, the energy harvested using the proposed films is sufficient to drive many small electronics and opens the route for this tactile system to be fully self‐powered in the future. All in all, this nanocomposite comprises a new route in terms of multifunctionality rationally targeted toward healthcare applications. Based on our *ex vivo* sphincter case study, we anticipate that this technology has the potential to improve detection of not only obstetric anal sphincter injury, but also a range of surgical applications in which contact/force/stiffness feedback is relevant and required.

As future work, we can envision the study of different representations of the force/stiffness data in the form of user‐friendly interfaces from a human‐computer interface (HCI) perspective. This may lead to a complete system comprising the sensorized glove and the interface to communicate real‐time force/stiffness information to the surgeons constructively, with the aim of improving the safety and training of microsurgical tasks. Force/stiffness perception is currently highly subjective and this system could lead to objective metrics that could serve as a foundation for safe and optimized procedures and training.

## 4 Experimental Section

### Materials

Acetone (ACS reagent, ≥99.5%, Sigma–Aldrich), Advanced Dulbecco's modified eagle medium (Advanced DMEM) (Thermofisher Scientific), antibiotic‐antimycotic (100×, Thermofisher Scientific), *N*,*N*‐dimethylformamide (DMF) (anhydrous, 99.8%, Sigma–Aldrich), DMSO (sterile filtered, TOCRIS), fetal bovine serum (FBS), GlutaMAXTM‐1 (200 mm 100×, Thermofisher Scientific), LB Agar (Invitrogen, powder (Lennox L agar), LB broth (Invitrogen, powder (Lennox L agar, Thermo Fisher Scientific), LIVE/DEADTM Viability/Cytotoxicity Kit (L3224, Thermofisher Scientific), MTT assay reagents (CyQUANTUM MTT Cell Viability Assay kit, Thermofisher Scientific), polyimide (PI) sheets (50 µm thickness, Sigma–Aldrich), Poly(methyl methacrylate) (PMMA) (average Mw ≈120 000 by GPC, Sigma–Aldrich), (Poly(vinylidene fluoride) (PVDF) (average Mw ≈530 000, pellets, Sigma–Aldrich), screen printable silver ink (DM‐SIP‐2005, Dycotec), Trypan blue solution (0.4%, Thermofisher Scientific), Trypsin‐EDTA (0.25%, Thermofisher Scientific), and zinc oxide (nanopowder, <100 nm particle size, Sigma–Aldrich) were all used as received.

### Synthesis of the Nanocomposite Coating

The triboelectric nanocomposite was formulated based on a fluoropolymer/acrylic blend^[^ ^67^ ^]^ to which nanoparticle fillers are added, resulting in a sprayable dispersion that can yield superhydrophobic coating.^[^ ^66^ ^]^ The fluoropolymer used was PVDF due to its dielectric^[^ ^67^ ^]^ and known biocompatible nature (Supporting Information S2). To overcome the poor adhesion of PVDF due to its hydrophobicity, it was blended with PMMA (an acrylic). The dielectric nanoparticle fillers (ZnO) were added to increase the surface roughness. The resulting nanostructured morphology is important since surface roughness enhances the contact area and thus triboelectrification.^[^ ^43^ ^]^ The process of nanocomposite formation was adapted from our previous work, which used cyanoacrylate‐based acrylic instead.^[^ ^68^ ^]^ Briefly, PMMA and PVDF were dissolved separately in DMF, creating 8 wt.% weight and 20 wt.% stock solutions, respectively. To dissolve the PVDF pellets, a temperature of 70 ^○^C was needed. The sprayable dispersion composed of 8.3% of the PVDF solution, 13.9% of the PMMA solution, 10% of ZnO nanoparticles and the balance being acetone (67.8%). The mixture was magnetically stirred for 1 h at room temperature, followed by bath sonication for 15 min and a second 15 min magnetic stirring at 1000 rpm to obtain a well‐dispersed composition before spraying directly on an electrode layer. An IWATA LPH‐80 (1 mm nozzle) spray gun was used to apply the coatings. The coated electrodes were annealed for 2 h at 125 ^○^C in open air.

### Fabrication of Sensorized Smart Surgical Glove

The electrodes and interconnects were directly screen printed on the glove; the ink used for this kind of printing has substantially low curing temperature (minimum 60 °C). The stencil designs were created using Eagle AutoCAD software. Following printing and curing of silver electrodes and interconnects, the triboelectric coating was sprayed on the fingertips using a custom stencil.

### Triboelectric Sensing/Detecting Setup

To characterize the triboelectric properties of the coating, first it was used in the standard freestanding triboelectric‐layer mode.^[^ ^72^ ^]^ This type of test consisted simply of moving from side to side (in a sliding fashion) a freestanding dielectric layer on two metal films, connected to an electronic load that served both as the counter triboelectric material and as the electrodes, as can be seen in Figure S1 (Supporting Information). This test effectively shows whether a film is triboelectric, since it would be creating electricity from charge transfer from friction. For the rest of the triboelectric tests, the sensor was fixed on a precision 3‐axis motorized stage (Aerotech, high resolution: <2 nm, repeatability: 75 nm, and accuracy: 250 nm) that was driven using G‐code. The assembly ensured testing with reproducible application of contact forces on the sensor. The contact probe was a 3D‐printed polylactide (PLA) mallet‐like plunger. The plunger tip area was 3 cm × 3 cm to cover the surface of the sensors. The plunger was fixed to an optical table, while the motorized stage‐mounted sensor was moved up and down to simulate “tapping” (normal contact) and then laterally to mimic the “rubbing” (shear contact). The open‐circuit voltage (VOC) was measured using an oscilloscope (MSO4032 Tektronix) with a 10 MΩ load in parallel with the sensor and the short‐circuit current (ISC) was measured by connecting either a digital multimeter (SDM3055) or Keithley's electrometer (6517B) in series.

### Force Calibration Setup

For sensor calibration, the test setup was extended to include 25‐N M4‐5U digital force gauge. The force gauge was mounted on the contact probe, so that when the sensor touched it, the force created was recorded by the gauge. The force gauge module and the electrometer module worked simultaneously by means of a virtual interface developed using LabView.

### Morphological characterization

Morphological characteristics of the coating were observed using a scanning electron microscope (SEM) (Zeiss, 1450XB). A small portion of the coating was directly placed on a carbon‐coated grid and then sputter coated with gold and observed at 20 kV. The thickness of the coating was also measured using the SEM by using a small portion of the single‐electrode sensor and placing it on double 90‐degree angled SEM aluminum stubs.

### Contact angle measurements

In order to characterize the superhydrophobicity of the surface, the advancing and receding water droplet contact angles were measured.^[^ ^41^, ^70^ ^]^ The procedure is described in more detail in Supporting Information S9.

### Cytotoxicity tests

The cells were cultured in DMEM supplemented with 10% FBS, 1% Antibiotic‐Antimycotic, and 2% GlutaMAX under standard cell culture conditions of 37°C and 5% CO2. A LIVE/DEAD cell viability assay kit (Thermofisher, L3224) was used to test cell viability by fluorescence imaging. Initially, small pieces of the nanocomposite coating were immersed separately in 4 mL of phosphate‐buffered saline (PBS) for 24, 48, and 72 h to obtain sample extracts. Next, 50 µL of sample extract (from each day) was added to 400 µL of DMEM media containing 5×104 cells/mL HDF cells in a 48‐well plate. After three days of incubation, the cells were washed with PBS and 400 µL LIVE/DEAD assay solution (prepared by mixing 5 µL of calcein AM (Component A) and 20 µL of ethidium homodimer‐1 (Component B) with 10 mL PBS) was added to each well and further incubated for 30 mins. Finally, the stained cells were observed directly using a fluorescence microscope (EVOS M5000). Biocompatibility of the sensor coating was further evaluated by MTT [3‐(4,5‐dimethylthiazol‐2‐yl)‐2,5‐diphenyltetrazolium bromide] assay. The procedures and concentrations of seeding cells were the same as the fluorescence imaging described above. Treated cells were washed three times with PBS and the medium was replaced with 180 µL fresh DMEM media followed by the addition of 20 µL of the MTT stock (5 mg mL^−1^) solution and further incubated for 4 h. The medium was then replaced with 200 µL DMSO and was incubated again for 20 min to dissolve the formazan crystals. A microplate plate reader (TECAN, 200 Pro, Switzerland) was used to measure the absorbance at 570 nm. Cell viability percentage was calculated with respect to control (without any sample). All experiments were repeated five times.

### Antibacterial tests

To facilitate clinical translation, tests with a series of bacterial strains commonly associated with maternal and neonatal sepsis were carried out to assess the antibacterial properties of the sensors. These strains were as follows: *E. coli* ATCC 25922, *S. aureus* ATCC 43300, and *S. agalactiae* (“Group B streptococcus”, GBS), which was obtained after informed patient consent from pregnant women booked at University College London Hospital NHS Foundation Trust with confirmed vaginal GBS infection as part of an ethically approved study called Treatment for Group B Streptococcal infection (IRAS Project ID: 196499, REC reference number: 16/LO/0941). Briefly, overnight‐grown cultures of bacteria (10^7^ CFU mL^−1^) were first plated on nutrient agar. Next, a small piece of the sample (15 mm × 15 mm—approximating a reasonable size of the fingertip area) coated with the nanocomposite (sensor material) was placed upside down at the center of the agar plate so that the coating is in direct contact with the agar. The plates were then incubated at 37 °C for 24 h before observing the zone of inhibition. This is a derivation of the Kirby–Bauer test carried out to assess the antimicrobial properties of nanocomposites.^[^ ^75^ ^]^

### Energy Harvesting Tests

The *V*_OC_ measurements were carried out in contact‐separation mode by manually tapping on a setup comprising two small boards connected to each other by means of four parallel springs.^[^ ^76^ ^]^ The triboelectric coating (2 cm × 2 cm) was placed on one of the boards while a layer of polyethersulfone (PES) (also 2 cm × 2 cm in size) was placed on the other board to make contact with the coating. PES was chosen due to its opposite rank with respect to our coating in the triboelectric series. The energy harvesting performance was additionally investigated using different load resistances (103–109 Ω). The effective power density was determined from:

(1) $$P=\frac{V^{2}}{A\times R_{L}}$$

where *A* is the effective surface area, *P* is the output power, *V* is the voltage, and *R*_L_ is the load resistance, respectively.

### Stiffness Change Tests

Both uncovered and covered sensors on the index fingertip of the glove were rubbed against either two, three, or four slots of different materials of the same height. Slots 1 and 3 are made out of ecoflex and slots 2 and 4 are made out of PDMS, with no change in height from one slot to the other. This was done by pouring and curing them on a 3D‐printed mold. These tests were performed manually by wearing the surgical sensorized glove. In Supporting Information S5, these tests were performed using the controlled setup and two different modes of contact (“tapping” and “rubbing”). For a second set of stiffness detection test in Supporting Information S5, three slots of three different materials (Teflon, PDMS and ecoflex) were used and they were manually rubbed with a covered sensorized glove. The interconnect coming from the index fingertip sensor was connected to the digital multimeter (SDM3055) that was in turn connected to the laptop. Signals were recorded using LabView.

### Pig Anal Sphincter Test Protocol

Pigs used for these studies were supplied by the Royal Veterinary College, Camden, London, as part of their tissue‐sharing resource. The protocol and scientific rationale for the supply and use was approved by the RVC's Ethical Review Board (request number 48 and approved on May 25, 2021). The pigs were humanely euthanized (pentobarbitone overdose) for a separate unrelated project. An obstetric Senior House Officer (SHO) tested the device ex vivo on an anal sphincter from a whole pig cadaver or after it had been dissected out to demonstrate repeatability and accurate detection of the sphincter defect. The sphincter defect was created by cutting the external sphincter at the 12 o'clock position using a scalpel similar to the position of a clinical obstetric anal sphincter injury. The interconnect coming from the index fingertip sensor was connected to Keithley's electrometer (6517B) which was connected to the laptop and signals were recorded using LabView.

## Conflict of Interest

The authors declare no conflict of interest.

## Supporting information

Supporting Information

Supplemental Video 1

Supplemental Video 2

Supplemental Video 3

Supplemental Video 4

Supplemental Video 5

Supplemental Video 6

## Data Availability

The data that support the findings of this study are available from the corresponding author upon reasonable request.
