# Supplementary material for: A Triboelectric Nanocomposite for Sterile Sensing, Energy Harvesting, and Haptic Diagnostics in Interventional Procedures from Surgical Gloves
Source: Adv Healthc Mater. 2023 Mar 11;12(17):2202673. doi: 10.1002/adhm.202202673 (PMC10614699; doi:10.1002/adhm.202202673)
Supplement: Supplementary file 1 — Supporting Information [file ADHM-12-2202673-s007.pdf]

ADVANCED
HEALTHCARE
MATERIALS

Supporting Information

for *Adv. Healthcare Mater.*, DOI 10.1002/adhm.202202673

A Triboelectric Nanocomposite for Sterile Sensing, Energy Harvesting, and Haptic Diagnostics in Interventional Procedures from Surgical Gloves

*Carmen Salvadores Fernandez, Shireen Jaufuraully, Biswajoy Bagchi, Wenqing Chen, Priyankan Datta, Priya Gupta, Anna L. David, Dimitrios Siassakos, Adrien Desjardins and Manish K. Tiwari**

Supporting Information

A triboelectric nanocomposite for sterile sensing, energy harvesting and haptic diagnostics in interventional procedures from surgical gloves

*Carmen Salvadores Fernandez, Shireen Jaufuraully, Biswajoy Bagchi, Priyankan Datta, Priya Gupta, Anna L. David, Dimitrios Siassakos, Adrien Desjardins, Manish K. Tiwari**

C. Salvadores Fernandez, Dr. S. Jaufuraully, Dr. B. Bagchi, Dr. P. Datta, Dr. P. Gupta, Prof. A. David, Prof. D. Siassakos, Prof. A. Desjardins, Prof. M. K. Tiwari

Wellcome/EPSRC Centre for Interventional and Surgical Sciences, UCL, London, W1W 7TS, UK

E-mail: m.tiwari@ucl.ac.uk

C. Salvadores Fernandez, Dr. B. Bagchi, Dr. P. Datta, Dr. P. Gupta, Prof. M. K. Tiwari

Nanoengineered Systems Laboratory, Mechanical Engineering, UCL, London, WC1E 7JE, UK

Dr. S. Jaufuraully, Prof. A. David, Prof. D. Siassakos

Elizabeth Garrett Anderson Institute for Women's Health, UCL, London WC1E 6AU

Prof. A. David, Prof. D. Siassakos

NIHR Biomedical Research Centre at UCL

Prof. A. Desjardins

Department of Medical Physics and Biomedical Engineering, University College London, Gower Street, London, WC1E 6BT, UK

1. Triboelectric properties

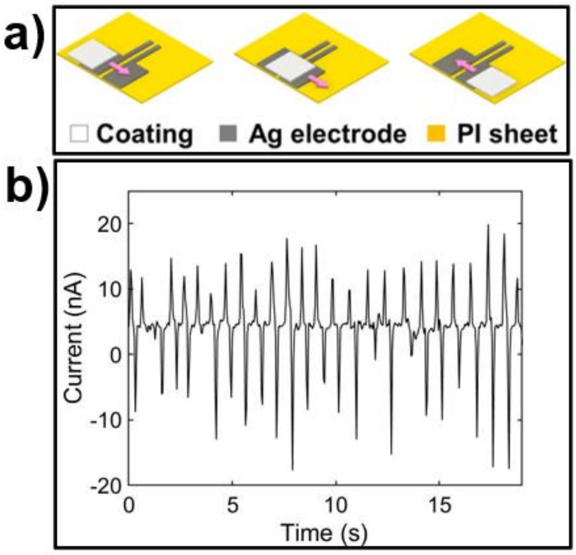

Figure S1. a) Schematic representation of the triboelectric basic freestanding rubbing test. b) Results obtained from the freestanding triboelectric-layer mode test.[1]

2. Material choice and regulations

The triboelectric coating was directly sprayed on the fingertip of surgical gloves. All the constituents used for these sensors are biocompatible and non-toxic to human tissue. The materials used for the synthesis of the sensors and which are exposed in the medical device are detailed below.

- Zinc oxide (ZnO) nanoparticles (nano powder, <100 nm particle size, Sigma Aldrich): The zinc oxide nanoparticles used for the coating were selected following the EU commission regulation standard 2016/621, and as covered in the assessment of this standard, pose no risk of adverse effects on human skin.[2–6] Zinc oxide nanoparticles' toxicity has been extensively studied and it has been established that when applied on the skin surface they do not penetrate nor cause toxicity in the viable epidermis.
- Poly(vinylidene fluoride) (PVDF) (average Mw ~530,000, pellets, Sigma Aldrich): From the PVDF's safety data sheet following EU regulation 1907/2006 it is classified as non-toxic and biocompatible.[7,8] Cytotoxicity tests have been carried out on PVDF for its use on medical devices, and it has shown no toxic effect.[9]
- Poly(methyl methacrylate) (PMMA) (average Mw ~120,000 by GPC, Sigma Aldrich): PMMA's cytotoxicity has been studied and the results show that when in contact with human tissues it does not present a biological threat and is non-toxic and biocompatible.[10–13]

The concentration of zinc oxide nanoparticles in the coating formulation was carefully selected following previous studies on nanoparticle filler effects in similar polymer blends.[14] Following the aforementioned study, the range of ZnO nanoparticle concentration which resulted in the highest contact angle (θ_A) measured was selected. Once it was established that this range of nanoparticle concentration was yielding superhydrophobic results following the contact angle tests described in **S9**, a 10% ZnO nanoparticle concentration was finally chosen for our triboelectric coating since it resulted in the highest power density (106 μW/cm^2).

3. SEM images and EDS of the triboelectric coating

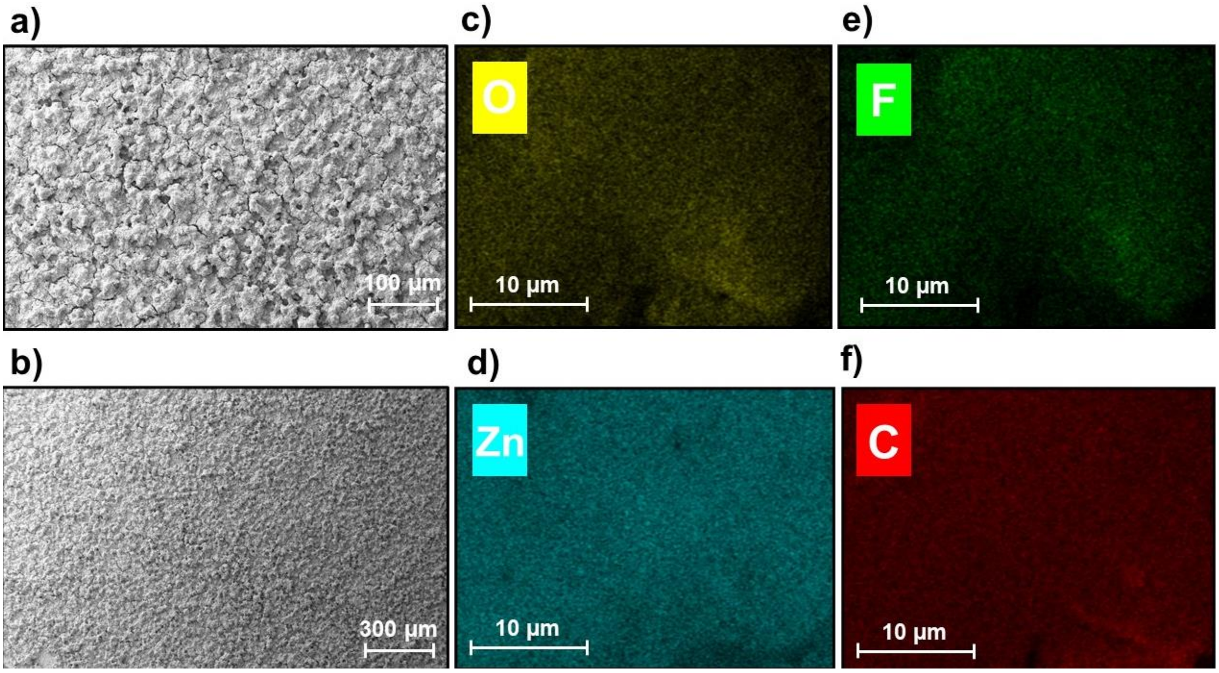

Figure S2. Scanning electron microscope **(**SEM) and Energy-dispersive X-ray spectroscopy (EDS) images of the triboelectric coating. a) SEM image of the triboelectric coating. b) SEM image of the triboelectric coating. EDS images showing distribution of c) Oxygen, d) Zinc, e) Fluorine and f) Carbon.

4. Enhancing piezoelectric film performance

The spray application of our triboelectric nanocomposite lends itself to versatility of applications. For example, they can be applied/used as a simple strategy to post-treat commercial sensors and to develop a prototype to demonstrate enhanced energy generation. This will add features to the sensing capabilities of the commercial film, enabling the detection two different modes of contact (tapping and rubbing). For proof of concept demonstration, the triboelectric coating was sprayed directly on commercial piezoelectric sensors electrodes **(Figure S3a)**. An LDT1-028K Piezo film was used to demonstrate the proof of concept. The coated film was annealed at 60 °C for 2 h. The leads on the commercial piezo film were used for circuit connections. After sensor fabrication, the test bench and procedure covered in the previous sections was again set up to measure open-circuit voltage (V_{OC}) and short-circuit current (I_{SC}). Both off-the-shelf piezoelectric sensor and the triboelectric-enhanced piezoelectric sensor prototypes were subjected to controlled tapping and rubbing contact to measure outputs experimentally. A digital signal-processing algorithm was implemented to filter noise and a full-wave bridge rectifier (shown in the supporting information) was set up to compare the temporal power density from the two sensors. A synergistic effect was found in both tapping and rubbing tests when the triboelectric layer was coated on the piezoelectric film, with clear enhancements in sensitivity. The triboelectric-enhanced prototype **(Figure S3b)** shows peak powers that are ca. twice that from the commercial piezoelectric sensors **(Figure S3c)**. Furthermore, it enables the detection of both normal (tapping) and shear (rubbing) contacts, as opposed to commercial piezoelectric films that only detect tapping. Commercial piezoelectric sensors can detect 'higher frequency' tapping contact; the triboelectric nanocomposite coating should extend its ability and enables capturing 'lower frequency' contacts such as the rubbing **(Figure S3d-e)**. Figure S3d shows five negative peaks, corresponding to the first contact of the test probe with commercial sensor during rubbing and approximately zero current for the rest of the test periods. Figure S3e also shows the five negative peaks, which remain the same, and the added response of the triboelectric-enhanced sensor featuring 10 nA constant current output. The current is induced by electron transfers from the coated surface to the top electrode of the commercial film.

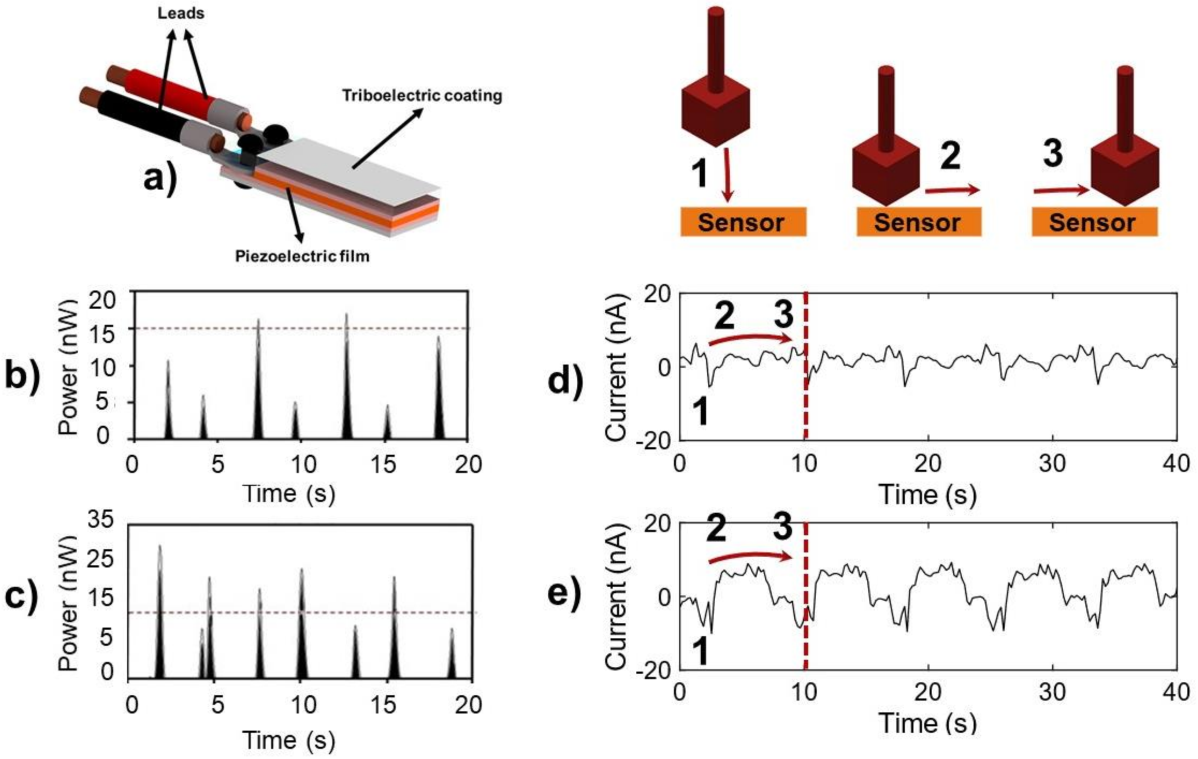

Figure S3. a) Temporal maximum power generated by tapping on the commercial piezoelectric sensor. b) Temporal maximum power generated by tapping on the triboelectric-enhanced piezoelectric sensor. c) Schematic of the triboelectric-enhanced piezoelectric commercial film prototype. d) Commercial piezoelectric sensor's response to controlled rubbing motion. e) Triboelectric-enhanced piezoelectric sensor's response to controlled rubbing motion.

5. Detection of stiffness change

The purpose of these tests was to use the nanocomposite sensors to distinguish changes in stiffnesses in different materials. In order to make sure that the measurements taken and the signal interpretations are not influenced by the relative position of the materials in contact with the sensor in the triboelectric series, the first tests were carried out with the sensor covered up by a surgical glove. The reasoning behind this was to not only decouple the influence of the materials position in the triboelectric series (since there would always be an isolating layer – the surgical glove - between the sensor and the materials it comes into contact with) from the influence of the materials stiffness, but also to establish the idea of feasible stiffness change detection even when the sensor is covered by a surgical glove. The controlled 'tapping' was carried out on 2 different materials (PDMS with a mixing ratio 10 - parts base elastomer and 1 - part curing agent and ecoflex) of different stiffnesses, PDMS being the stiffest and ecoflex the softest. Next, rubbing mode was used. The covered sensors were rubbed against 2 slots of different materials of the same height. Slots 1 and 3 are made out of ecoflex and slots 2 and 4 are made out of PDMS, with no change in height from one slot to the other. This was done by pouring and curing them on a 3D-printed mould. These tests were performed both using the controlled motorized setup described in **Section 4**. For the tapping tests (**Figure S4a**), we can see that once again the current peak's slope is larger for the softer materials, as demonstrated in **Section 2.1**. When performing a rubbing test with the controlled setup, this change in slope can be clearly seen in **Figure S4b** when crossing from ecoflex (softer and thus steeper response) to PDMS.

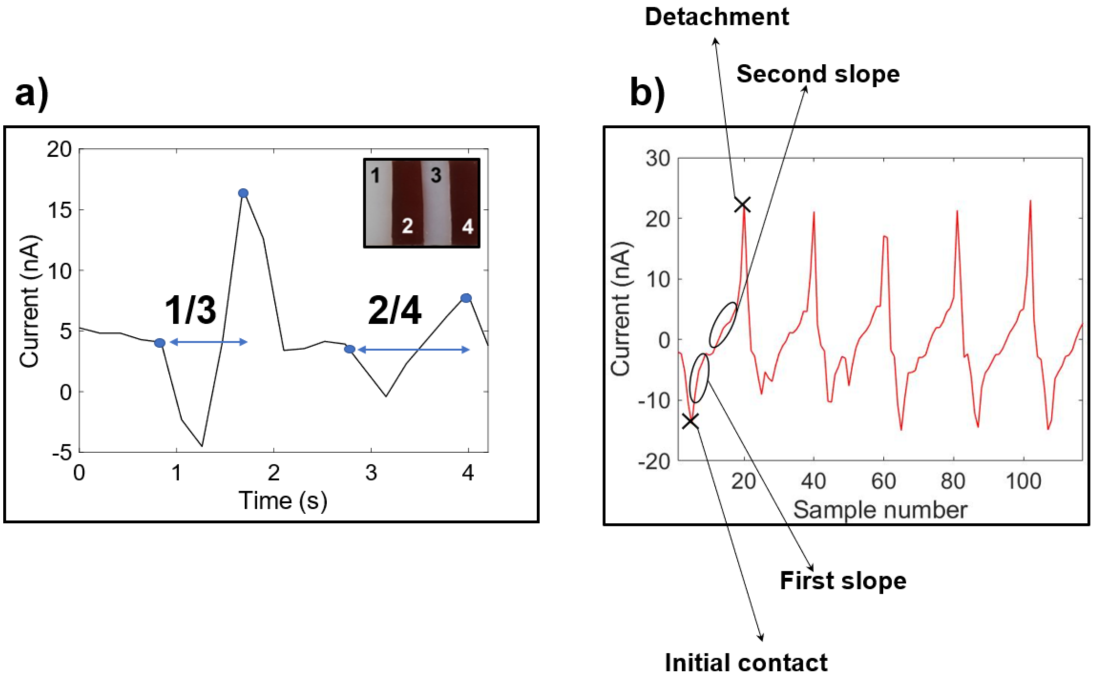

Figure S4. Change in stiffness tests using the 4 slots and controlled motorized stage setup described in **Section 4**. a) Tapping test performed on slots 1 and 3 (ecoflex) and 2/4 (PDMS) showing larger peak width for the harder material. b) Rubbing test performed through slots 1 to 2 (ecoflex to PDMS).

Next, a manual test was performed by wearing the surgical sensorised glove. For this second set of stiffness detection test, we used 3 slots of 3 different materials (Teflon, PDMS and ecoflex) and manually rubbed across them with a covered sensorised glove. This test is no longer in a controlled and repeatable environment (such as the previous test shown in **S3** and the force tests shown in **Section 2.2**) and as a result, current peaks begin to appear when crossing from one material of different stiffness to another. This behaviour of the sensors is further described in **Section 2.7** and **Section 2.8**.

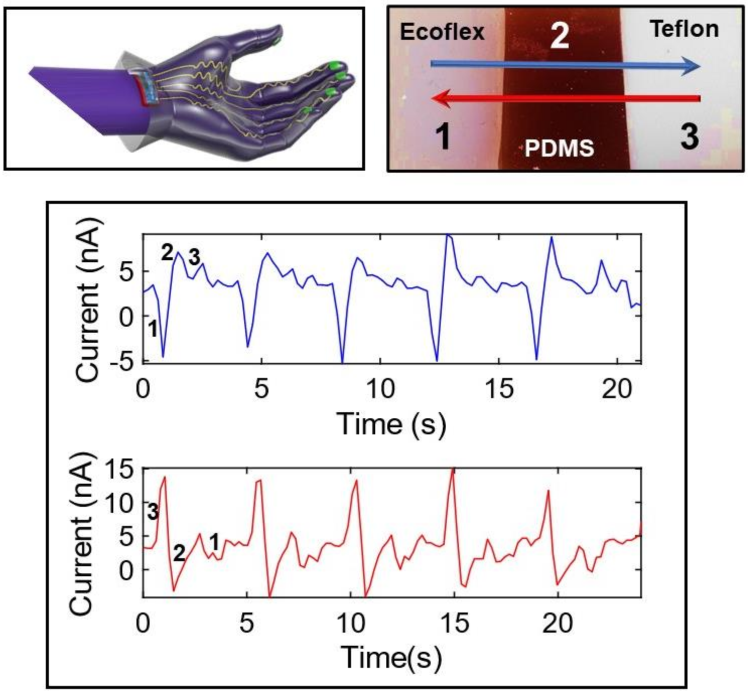

Figure S5. Comparison of peaks produced by rubbing against the 3 different slots shown in the top right using the sensorised glove covered up by a surgical glove (from left to right – blue signal, from right to left – red signal).

6. *Ex vivo* anal sphincter detection tests results

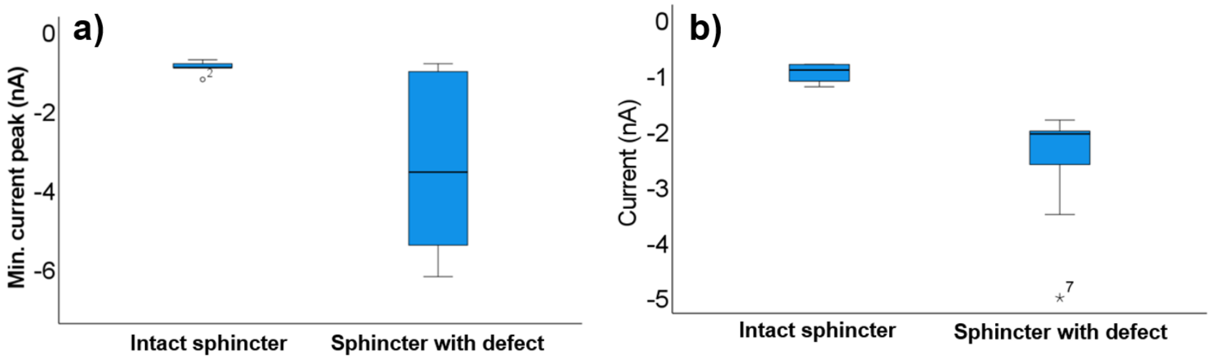

Figure S6. Median minimum current peak obtained throughout the anal sphincter detection tests on the intact sphincter vs. sphincter with a defect. a) Tests carried out on the dissected pig's anal sphincter. b) Tests carried out on the pig cadaver's anal sphincter.

7. Incorporation of the coating onto the surgical glove

A minimum number of spray passes is desirable to keep thickness as low as possible. However, lower number of passes may result in uneven coating thickness and an erratic/variable sensor output current. Thus, we analysed the effect of coating thickness on sensor output (see Figure S6). Each box plot in **Figure S6** shows the variability and magnitude of the current peaks recorded from 30 repeatable tapping tests for each coating sample shown (with different number of spray passes). This may be due to microscopically non-uniform coating thickness and the fact that crack formation due to any defect present can quickly reach the electrode and expose it to the contacting object. Once a threshold number of passes and coating thickness are surpassed, the current output and roughness of the coating (**Figure S6** and **Figure S7**) stabilise. This is advantageous for consistent detection of slots of different materials and anal sphincter injury in ex vivo studies. Following this, with progressive increase in the number of passes or thickness (beyond 12 passes in **Figure S6**), the coatings will be expected to become stiff and flaky, which may explain lower or less stable current outputs, and greater chances of coating delamination. The reduced flexibility of the coating together with the decrease in roughness (as shown in **Figure S7**), should also lead to a lower contact area and deformation. This can explain the reduction in the magnitude of the sensor current peaks. Overall, this suggests an optimal range of spray passes for which the triboelectric properties and robustness are most suitable to be 6 – 10 spray passes.

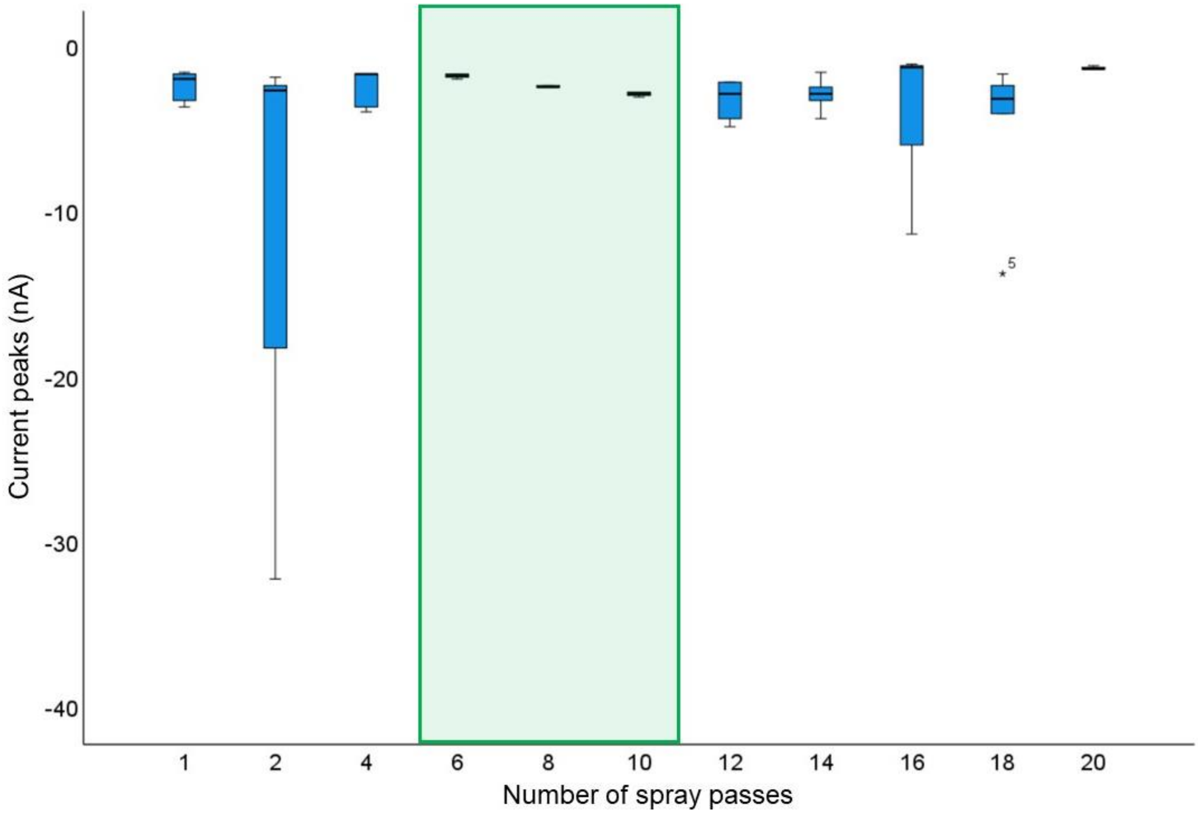

Figure S7. Box plot showing the variability and magnitude of the current peaks recorded when carrying out the repeatable tapping test. Each box plot is generated from 30 different set of measurements on coatings with a given number of spray passes. The area highlighted in green shows the optimal range of spray passes.

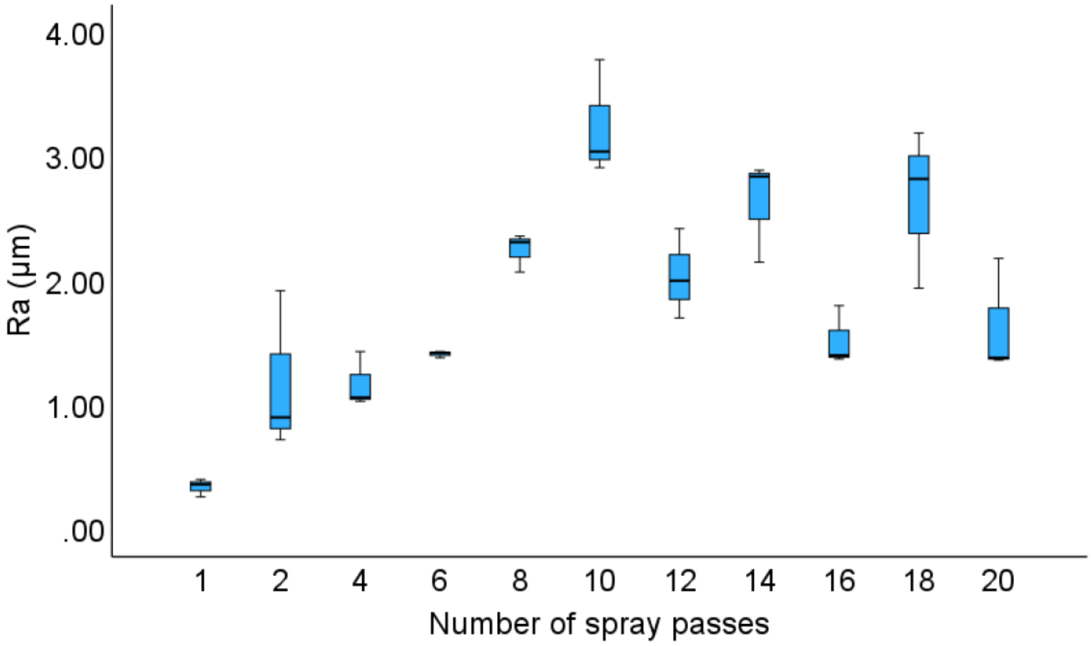

Figure S8. Box plot showing the variability and magnitude of the measured roughness values for each PVDF-PMMA-ZnO coating with increasing number of spray passes.

8. Coating adhesion and robustness

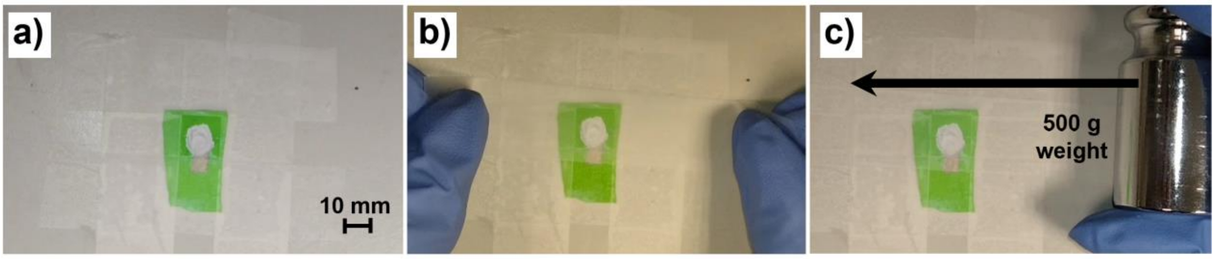

Figure S9. Tape peel test and results. a) Printed electrode (grey) and sprayed triboelectric coating (white) on a segment of a surgical glove (green). b) The tape was firmly pressed on the coating. c) 500 g weight roller used to apply pressure for the adhesion test.

Bonding tape (3M, with an adhesion to steel value of 2.5 N cm-1, commonly employed in standard tape peel tests) was used to test the adhesion of the coating. The protocol involved applying the tape to the coating by rolling a 500 g steel roller over it, as shown in **Figure S9a-c**. After a 90 s wait, the tape was peeled off, completing one repetition of the testing process. A new piece of tape was used for each peel-off cycle.[15] The triboelectric current output was recorded following each tape peel test using the repeatable tapping (see **Section 4** of the manuscript - *Triboelectric sensing/detecting setup*) 30 times. The result is plotted in **Figure S10**; the sensor (coating) is able to sustain satisfactory performance for at least 20 tape peel cycles. Following 25 cycles, the output variability began to rise slightly increase. Nevertheless, it remained within acceptable limits, and showed no signs of delamination (as shown in **Video S6**).

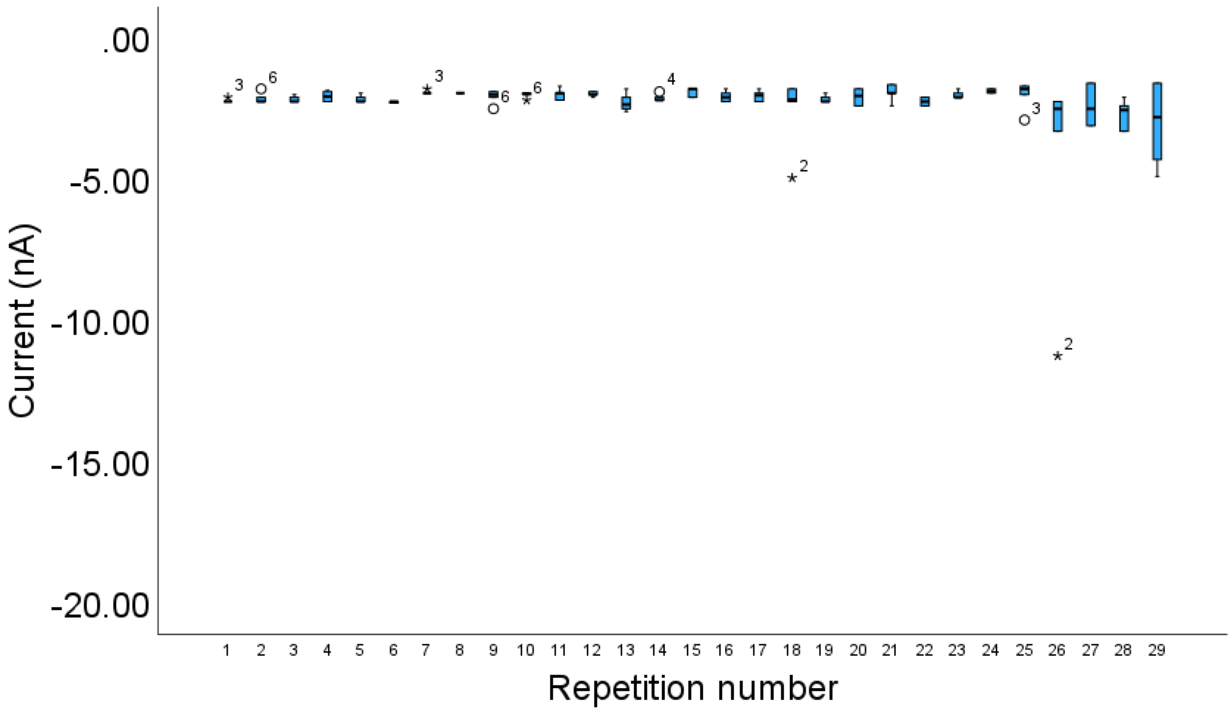

Figure S10. Box plot showing the variability and magnitude of the current peaks recorded when carrying out a repeatable tapping test 30 times with the coating after each repetition of the tape test protocol.

9. Contact angle measurement

The test is carried out by first setting up the camera, lights and syringe.[15] Next, the camera software is opened and the camera is focused in order to optimize the resulting image. The flow rate of the syringe must be set at about 0.4 mL/minute, in order to have a good control of the experiment. Water can be then pumped towards the film surface sample on which the camera is focused. When the drop is close to the surface, it is necessary to start recording, in order to take screenshots of the advancing and receding droplet. In order to do so, the pump must be stopped and the drop be withdrawn when it has stopped growing. Next, the MATLAB programme enables the use of a photo taken using the setup shown in **Figure S11** and, after determining specific points of the boundary of the drop, gives the contact angle result.[16]

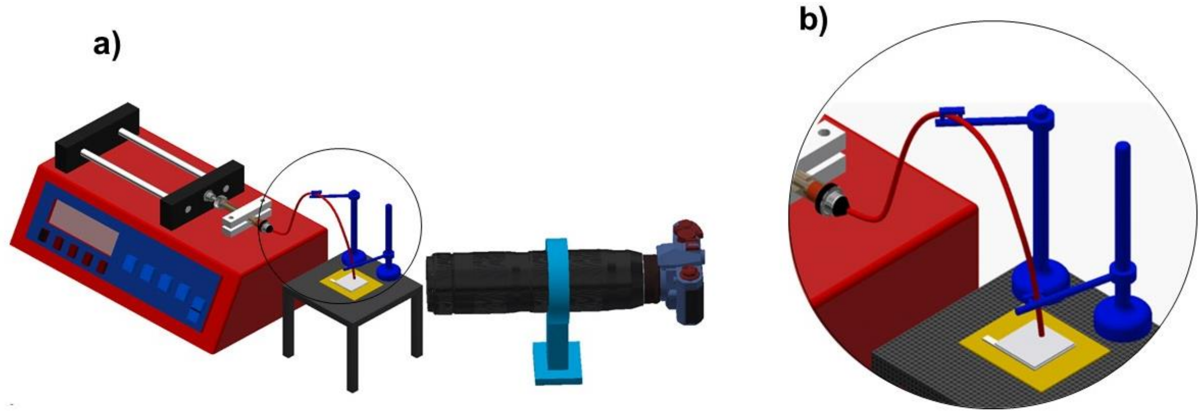

Figure S11. Schematic illustration of the contact angle measurement setup. a) Schematic illustration of the whole setup (with syringe pump assembly, sample stage and a CMOS camera). b) Close up schematic illustration of the stage with the sample and the water flow tube.

Videos Supporting Information

Video S1

Video demonstrating that the output of the triboelectric nanocomposite coating can be used to light up to 50 commercial blue LEDs.

Video S2

Video showing a repetition of the stiffness change detection test wearing the sensorised glove. As can be seen on the multimeter's screen, negative peaks form when the sensor makes contact with a given material, and similarly positive peaks are formed when contact is released from the material. The change in the current signal when going from one material to the other discussed in **Section 2.6** of the manuscript can also be observed.

Video S3

Video of obstetrician explaining the clinical background regarding the difficulties surrounding vaginal examinations in operative vaginal birth and the importance of accurate fetal position assessment. In the second half of the video, the way in which we have aimed to tackle this issue with the sensorised glove is also covered.

Video S4

Video showing obstetrician wearing the sensorised surgical glove covered by a second surgical glove carrying out a vaginal examination on the neonatal phantom we have developed in our lab. As demonstrated in the video, the sensorised glove is able to accurately assess fetal position in the neonatal phantom. It is able to reliably detect fetal sutures and as a result our user-friendly interface (created using LabView) successfully displays a green triangle when encountering the posterior fontanelle and a red diamond when encountering the anterior fontanelle.

Video S5

Video displaying obstetrician carrying out the obstetric anal sphincter detection test on an anal sphincter in a non-dissected whole pig cadaver wearing the sensorised glove covered by a second surgical glove. As can be seen in the user-friendly interface that we have developed using LabView, the sensor is accurately detecting the defect.

Video S6

Video presenting the tape peel test and additional robustness demonstrations using a blade and a pair of scissors.

References

[1] Z. L. Wang, L. Lin, J. Chen, S. Niu, Y. Zi, *Triboelectric Nanogenerators (Green Energy and Technology)*, Springer, Switzerland, **2016**.

[2] SCCS (Scientific Committee on Consumer Safety), **2014**.

[3] SCCS (Scientific Committee on Consumer Safety), *Opinion SCCS/1489/12* **2012**, *COLIPA S 7*, 112.

[4] SCCP (Scientific Committee on Consumer Products), **2005**.

[5] European Commission, **2018**, *2016*, 48.

[6] SCCNFP (Scientific Committee on Cosmetics Products and Non-Food Products), **2003**.

[7] Gujarat, Fluorochemials, Inoflar, *PVDF (Polyvinylidene Fluoride Homopolymer),* **2018**.

[8] B. Gmbh, V. Bohlender, S. Tauberfranken, *Safety Data Sheet PVDF*, **2016**, *2006*.

[9] F. J. C. Braga, S. O. Rogero, A. A. Couto, R. F. C. Marques, A. A. Ribeiro, J. S. de C. Campos, *Materials Research* **2007**, *10*, 247.

[10] A. P. Bastidas-Coral, A. D. Bakker, C. J. Kleverlaan, J. M. A. Hogervorst, J. Klein-Nulend, T. Forouzanfar, *J Biomed Mater Res B Appl Biomater* **2020**, *108*, 1536.

[11] L. Z. Linan, N. M. N. Lima, C. Benatti, M. Xavier, A. A. Rodrigues, F. Manenti, A. Jardini, R. M. Filho, R. Gilioli, *Brazilian Archives of Biology and Technology* **2018**, *61*, 1.

[12] E. Commission, *European Union Risk Assessment Report*, **2002**.

[13] O. S. Manoukian, N. Sardashti, T. Stedman, K. Gailiunas, A. Ojha, A. Penalosa, C. Mancuso, M. Hobert, S. G. Kumbar, in *Encyclopedia of Biomedical Engineering*, Elsevier, **2019**, pp. 462–482.

[14] M. K. Tiwari, I. S. Bayer, G. M. Jursich, T. M. Schutzius, C. M. Megaridis, *ACS Appl Mater Interfaces* **2010**, *2*, 1114.

[15] C. Peng, Z. Chen, M. K. Tiwari, *Nat Mater* **2018**, *17*, 355.

[16] M. Grizen, T. Maitra, J. P. Bradley, M. K. Tiwari, *Heat Transfer Engineering* **2020**, *41*, 1663.
